# *In vivo* efficacy of combination of colistin with fosfomycin or minocycline in a mouse model of multidrug-resistant *Acinetobacter baumannii* pneumonia

**DOI:** 10.1038/s41598-019-53714-0

**Published:** 2019-11-20

**Authors:** Nam Su Ku, Su-Hyung Lee, Young- soun Lim, Heun Choi, Jin Young Ahn, Su Jin Jeong, Sung Jae Shin, Jun Yong Choi, Young Hwa Choi, Joon-Sup Yeom, Dongeun Yong, Young Goo Song, June Myung Kim

**Affiliations:** 1 Department of Internal Medicine, Yonsei University College of Medicine, Seoul, South Korea; 2 AIDS research Institute, Yonsei University College of Medicine, Seoul, South Korea; 3 Branch of Carcinogenesis and Metastasis, Research Institute of National Cancer Center, Goyang-si, South Korea; 4 Department of Microbiology, Institute for Immunology and Immunological Diseases, Brain Korea 21 PLUS Project for Medical Science, Yonsei University College of Medicine, Seoul, South Korea; 5 Department of infectious diseases, Ajou University School of Medicine, Suwon, Korea; 6 Department of Laboratory Medicine and Research Institute of Bacterial Resistance, Yonsei University College of Medicine, Seoul, South Korea

**Keywords:** Microbiology, Bacterial infection

## Abstract

Unfortunately, the options for treating multidrug-resistant (MDR) *Acinetobacter baumannii* (*A*. *baumannii*) infections are extremely limited. Recently, fosfomycin and minocycline were newly introduced as a treatment option for MDR *A*. *baumannii* infection. Therefore, we investigated the efficacy of the combination of colistin with fosfomycin and minocycline, respectively, as therapeutic options in MDR *A*. *baumannii* pneumonia. We examined a carbapenem-resistant *A*. *baumannii* isolated from clinical specimens at Severance Hospital, Seoul, Korea. The effect of colistin with fosfomycin, and colistin with minocycline on the bacterial counts in lung tissue was investigated in a mouse model of pneumonia caused by MDR *A*. *baumannii*. *In vivo*, colistin with fosfomycin or minocycline significantly (*p* < 0.05) reduced the bacterial load in the lungs compared with the controls at 24 and 48 h. In the combination groups, the bacterial loads differed significantly (*p* < 0.05) from that with the more active antimicrobial alone. Moreover, the combination regimens of colistin with fosfomycin and colistin with minocycline showed bactericidal and synergistic effects compared with the more active antimicrobial alone at 24 and 48 h. This study demonstrated the synergistic effects of combination regimens of colistin with fosfomycin and minocycline, respectively, as therapeutic options in pneumonia caused by MDR *A*. *baumannii*.

## Introduction

*Acinetobacter baumannii* is a well-documented, multidrug-resistant (MDR) nosocomial pathogen^1^. In the past, carbapenems have been recommended as the antibiotics of choice for treating *A*. *baumannii*. However, the increasing incidence of MDR strains has led to use of unconventional antibiotics, such as polymyxin, rifampicin, and tigecycline, for the treatment of MDR isolates^2^. Nevertheless, pneumonia caused by MDR *A*. *baumannii* infection has a high mortality rate^3^.

Colistin has been used increasingly for the treatment of MDR *A*. *baumannii* infection, despite its potential nephrotoxicity and neurotoxicity. It has shown excellent *in vitro* antibacterial effect against carbapenem-resistant *A. baumannii*^4^. However, low plasma concentrations, heteroresistance and rapid regrowth after colistin treatment have brought the efficacy of colistin monotherapy into question^5^.

Consequently, colistin-based combination treatments have been proposed to attain antibiotic synergy. A systematic review revealed that colistin-based combinations with several antibiotics exerted a synergistic effect against isolates of MDR *A*. *baumannii* and lowered the mortality rate in animal studies^6–11^.

Recently, minocycline and fosfomycin, two ‘old’ drugs, were newly introduced as treatment options for MDR *A*. *baumannii* infection. Minocycline had the second highest susceptibility rate (79.1%), followed by colistin *in vitro*^12^. Fosfomycin is active against Gram-positive and -negative bacteria^13^. Also, fosfomycin is an alternative agent for the treatment of MDR bacterial infections^14,15^.

However, to our literature search, little *in vivo* data are available on the use of colistin with minocycline or fosfomycin for the treatment of infection by MDR *A*. *baumannii*, especially pneumonia. Therefore, we investigated the *in vivo* efficacy of combinations of colistin with minocycline and fosfomycin, respectively, as therapeutic options in a mouse model of MDR *A*. *baumannii* pneumonia.

## Results

### Antibiotic susceptibility tests

Table 1 shows the minimum inhibitory concentrations (MICs) of piperacillin/tazobactam, ceftazidime, imipenem, amikacin, ciprofloxacin, tigecycline, colistin, minocycline, and fosfomycin of an *A. baumannii* isolate.

**Table 1:** Antibiotic susceptibility of carbapenemase–producing *A*. *baumannii*.

β-lactamase^*^	MIC(mg/L)								
	PIP/TAZ	CAZ	IMP	AMK	CIP	TIG	COL	MIN	FOS
OXA-23	>128	>128	32	>128	>128	16	0.5	0.25	>128

MIC, minimal inhibitory concentration; PIP/TAZ, piperacillin/tazobactam; CAZ, ceftazidime; IMP, imipenem; AMK, amikacin; CIP, ciprofloxacin; TIG, tigecycline; COL, colistin; MIN, minocycline; FOS, fosfomycin.

^*^There were no additional chromosomal beta-lactamase genes in this strain.

### Time-kill test

**Figure 1:**
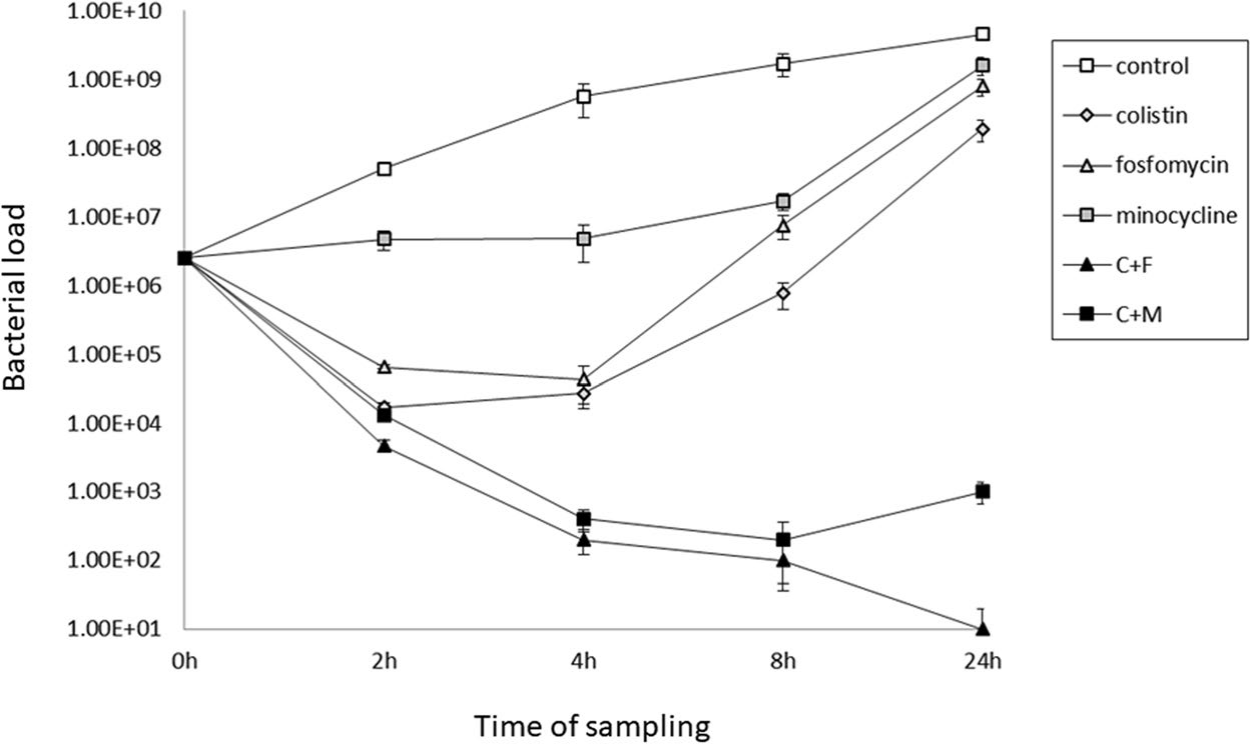
Time-kill curves. Colistin/fosfomycin and colistin/minocycline combinations showed bactericidal effects, with >3 log_10_ reductions in CFU at 4, 8, and 24 h. The combination regimens showed synergistic effects, with ≥2 log_10_ decreases in CFU compared with the monotherapy regimens (0.5 × MIC). (C: colistin; F: fosfomycin; M: minocycline).

At 0.5 × MIC, combinations of colistin with fosfomycin and colistin with minocycline showed synergistic effects, with >2 log _10_ reductions in CFU at 4 and 8 h. Moreover, this reduction in CFU was maintained at 24 h. The combination regimens showed synergistic effects with ≥2 log_10_ decreases compared with the monotherapy regimens. The time–kill curves are shown in Fig. 1.

### Mouse model of MDR *A*. *baumannii* pneumonia

**Figure 2:**
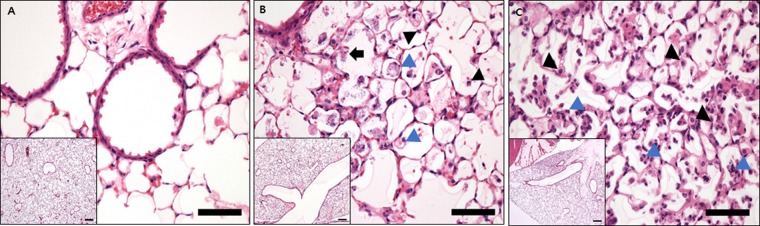
Histopathology of the lung tissues of mice at 4 (**A**), 24 (**B**), and 48 h (**C**) after inoculation with *A*. *baumannii* (H&E; bar = 100 µm). (**A**) At 4 h after inoculation, no significant lesion was present in the alveoli, bronchioles, or bronchi, except for minimal edema in the alveolar cavity. (**B**) At 24 h after inoculation, moderate numbers of neutrophils (dark triangle) and macrophages (blue triangle) had infiltrated the alveoli; moderate edema was present, and bacterial colonization (dark arrow) was observed in the alveolar cavities. (**C**) At 48 h after inoculation, large numbers of neutrophils (dark triangle) and macrophages (blue triangle) had infiltrated the alveoli.

Figure 2 shows the lung pathology at 4, 24, and 48 h after nasal inoculation. Four hours after inoculation, no significant lesion was present in the alveoli, bronchioles, or bronchi, except for minimal edema in the alveolar cavity. At 24 h after nasal inoculation, moderate numbers of neutrophils and macrophages had infiltrated the alveoli, with moderate edema, and bacterial colonization was observed in the alveolar cavities. At 48 h after nasal inoculation, large numbers of macrophages and neutrophils had infiltrated the alveoli.

### Effects on lung bacterial loads

Table 2 shows the lung bacterial loads in each group. Colistin, fosfomycin, and minocycline significantly (*p* < 0.05) reduced the bacterial loads in the lungs, compared with the controls, at 24 and 48 h. At 24 h after starting the antibiotic agents, fosfomycin and minocycline showed bactericidal effects, but colistin did not. However, colistin in combination with fosfomycin and minocycline, respectively, significantly (*p* < 0.05) reduced the bacterial load in the lungs compared with the controls at 24 and 48 h. In the combination groups, significant (*p* < 0.05) differences were noted in the bacterial loads compared with the more active antimicrobial alone. Moreover, the combination regimens all showed bactericidal and synergistic effects at 24 and 48 h compared with the more active antimicrobial alone. The combination of colistin with fosfomycin significantly (*p* < 0.05) reduced the bacterial load in the lungs at 48 h compared with colistin with minocycline.

**Table 2:** Therapeutic effects on the lung bacterial loads at 24 and 48 h after starting the antibiotic agents.

Antibiotic regimen	24 h	48 h
Control	12.24 ± 0.44	12.99 ± 0.22
COL	9.65 ± 0.43^a^	8.73 ± 0.34^a^
FOS	7.68 ± 0.47^a^	6.68 ± 1.02^a^
MIN	9.26 ± 0.19^a^	7.73 ± 1.33^a^
COL + FOS	5.63 ± 0.26^a,b^	3.46 ± 0.42^a,b,c^
COL + MIN	6.07 ± 1.04^a,b^	5.16 ± 0.83^a,b^

COL, colistin; MIN, minocycline; FOS, fosfomycin.

The lung bacterial loads are expressed as the mean ± standard deviation of log_10_ CFU per gram of lung at 24 and 48 h.

^a^Significant difference in bacterial load compared with the control group (*p* < 0.05).

^b^Significant difference in bacterial load compared with the more active antibiotic alone (*p* < 0.05).

^c^Significant difference in bacterial load compared with the other combination (*p* < 0.05).

### Effects on survival

Table 3 shows the mortality rates of the mice. The mortality rate was 33.3% (4/12) in the untreated control group at 48 h. No significant difference in survival was observed among the control and antibiotic-treated groups.

**Table 3:** Mortality rates in the control and antibiotic-treated groups.

Antibiotic regimen	24 h	48 h
Control	0/15 (0%)	4/12 (33.3%)
COL	0/15 (0%)	0/12 (0%)
FOS	0/15 (0%)	0/12 (0%)
MIN	0/15 (0%)	0/12 (0%)
COL + FOS	0/15 (0%)	0/12 (0%)
COL + MIN	0/15 (0%)	0/12 (0%)

COL, colistin; MIN, minocycline; FOS, fosfomycin.

## Discussion

This study demonstrated the synergistic effects of regimens combining colistin with minocycline and fosfomycin, respectively, on pneumonia caused by MDR *A*. *baumannii*.

The treatment of *A*. *baumannii* infection is an important problem in the nosocomial setting^16^. In serious infections including pneumonia, initial therapy with an appropriate antibiotic is very important^6^. In addition to colistin, other antibiotic regimens have emerged, although none has been fully tested. Carbapenem with sulbactam showed a synergistic effect against in MDR *A*. *baumannii* strains^6,7^. Although the study population was heterogeneous and small and no control group was used, a clinical study of rifampicin in combination with colistin and imipenem, yielded promising results^8,9^. Tigecycline also showed good bacteriostatic activity against carbapenem-resistant *A*. *baumannii in vitro*^6,10^. However, the synergistic activity of many antibiotics *in vitro* does not correlate with that *in vivo*^17^. Nevertheless, some specific antibiotic combinations have shown increased *in vivo* efficacy against MDR isolates^18^.

Recently, fosfomycin and minocycline were introduced as treatment options for the infection caused by MDR *A*. *baumannii*^12,13^. In our time–kill study, colistin with fosfomycin and minocycline, respectively, showed bactericidal and synergistic effects at 8 and 24 h. A previous study using time–kill tests documented synergistic and bactericidal effects of colistin and minocycline in 92% of the strains tested at 24 h^2^. In another study using the E-test, the fractional inhibitory concentration indices (FICIs) for combinations of polymyxin B and minocycline were generally ≤0.5 or >0.5–1.0, suggesting that polymyxin B and minocycline have a synergistic or additive effect^19^. In the same study, most FICIs for polymyxin B and fosfomycin were within the ranges of 0.5–1.0 and 1.0–4.0, suggesting that polymyxin B and fosfomycin exert an additive or independent effect^19^. In comparison, another study of combination therapy against *A*. *baumannii* found that colistin combined with fosfomycin was more effective than colistin monotherapy in 83.3% (24 h) and 66.7% (48 h) of MDR strains^18^.

We also found that colistin with minocycline and colistin with fosfomycin showed bactericidal and synergistic effects 24 and 48 h after nasal inoculation *in vivo*, in accordance with previous studies. Yang *et al*.^20^ reported that minocycline in combination with colistin had *in vivo* synergistic efficacy against MDR A. *baumannii* pneumonia. Bowers *et al*.^21^ showed that minocycline combined with polymyxin B further decreased the bacterial lung load at 24 h, compared with monotherapy. Sirijatuphat *et al*.^22^ reported that colistin with fosfomycin showed a synergistic effect against carbapenem-resistant *A*. *baumannii*. They also recently conducted a preliminary clinical study, which showed that patients with MDR *A*. *baumannii* infection given a combination of colistin and fosfomycin had significantly better microbiological responses with trends toward more favorable treatment outcomes and lower mortality compared with those treated with colistin alone^23^. However, because several types of infection, polymicrobial infections and concurrent antimicrobial agents were included in their study, it was heterogenous. Although our study was an *in vivo* animal study, not human study, it was more homogenous than previous study.

Interestingly, our *in vivo* results showed that the combination of colistin with fosfomycin significantly reduced the bacterial load in the lungs, compared with monotherapy and colistin with minocycline, at 48 h. Fosfomycin has a higher MIC against *A*. *baumannii*. For an antibiotic to be effective clinically, it must achieve concentrations in the interstitial fluid that exceed the MICs for the pathogens^24,25^. One study showed that fosfomycin achieved antimicrobially effective concentrations in infected lung tissue^26^. Also, without pharmacokinetics (PKs) of fosfomycin in this study, we used it every 4 h according to other study. These frequent injections of fosfomycin might attain more effective concentrations in infected lung tissue than minocycline. Moreover, in the combination therapy, fosfomycin was injected with colistin simultaneously, but minocycline was not. This difference explains the greater effectiveness of colistin with fosfomycin relative to colistin with minocycline at 48 h.

No significant difference in survival was observed among the control and antibiotic-treated groups. This finding might be due to the relatively short duration of follow-up in our study or the relatively low virulence of OXA-23 carbapenemase-producing *A. baumannii*. According to a previous report, OXA-23 carbapenemase-producing *A. baumannii* infection has a high mortality rate in intensive care unit patients^27^. Consequently, further evaluation of the mortality rate of OXA-23 carbapenemase-producing *A. baumannii* infection with a longer follow-up is needed.

Our study has some limitations. First, only one strain from a single center was used. However, we used an OXA-23 carbapenemase-producing *A. baumannii* isolated from clinical specimens obtained in our hospital. OXA-23 carbapenemase is the most common carbapenemase in South Korea^28^. Therefore, our study was meaningful in this regard. Second, we did not investigate the PKs of the drugs used in this study. However, we used dosages that have been used in other studies.

In conclusion, we investigated the *in vivo* efficacy of colistin in combination with minocycline and fosfomycin, as therapeutic options in a mouse model of MDR *A*. *baumannii* pneumonia. We demonstrated the *in vivo* synergistic effects of regimens combining colistin with minocycline and fosfomycin, on pneumonia caused by MDR *A*. *baumannii*. Large clinical trials are needed to clarify the role of regimens combining colistin with fosfomycin or minocycline in treating MDR *A*. *baumannii* pneumonia.

## Methods

### Bacterial strains

We obtained five carbapenem-resistant *A*. *baumannii*, which have an OXA-23 carbapenemase, isolated from clinical specimens obtained at Severance Hospital, Seoul, South Korea. We selected the most virulent strain, which was isolated from a patient with pneumonia and bacteremia. The other strains were colonizers isolated from sputum specimens. A strain was considered resistant to carbapenems when the MIC against imipenem was ≥16 mg/L.

### Antibiotic susceptibility tests

Antibiotic susceptibility tests were performed in duplicate using the agar dilution and broth microdilution methods according to the Clinical and Laboratory Standards Institute (CLSI)^29^. The MICs of piperacillin/tazobactam, ceftazidime, imipenem, amikacin, ciprofloxacin, minocycline, and fosfomycin were determined by the agar dilution method. The MICs of colistin were determined by the broth microdilution method. The MICs of fosfomycin were determined by the agar dilution method in cation-adjusted Mueller-Hinton medium, supplemented with 25 mg/L glucose-6-phosphate (Sigma-Aldrich., St. Louis, MO, USA). Because there are no CLSI interpretive criteria for fosfomycin against *A. baumannii*, the fosfomycin breakpoints for *Escherichia coli* were used according to the CLSI guidelines^29^. *E. coli* ATCC 25922 and *Pseudomonas aeruginosa* ATCC 27853 were used for quality control of antimicrobial susceptibility testing.

### Detection of carbapenemase genes by PCR

After DNA extraction multiplex PCR was performed to detect blaOXA genes (blaOXA-23-like, blaOXA-24-like, and blaOXA-58-like genes) and ISAba1-associated blaOXA-51-like gene^30^. Additionally, PCR was performed to detect blaOXA-182 gene^31^. We performed PCR to detect blaIMP-1-like, blaVIM-2-like, and blaSIM-1-like genes^32^. Sequence analysis was performed by a commercial laboratory (Macrogen, Seoul, South Korea).

### Time–kill test

For both agents and their combinations, time–kill tests were performed using sub-inhibitory For both agents and their combinations, (0.5 × MIC)^33^. Ten-fold dilutions were inoculated onto Mueller–Hinton agar and colonies were counted at 0, 4, 8, and 24 h. Bactericidal activity was defined as a ≥3 log_10_ decrease compared with the initial inoculum. Synergy was defined as a ≥2 log_10_ decrease with the combination, compared with most active single agent^34^. Experiments were performed in triplicate on separate days. Results were read by two observers.

### Mouse model of MDR *A*. *baumannii* pneumonia

The animal study was approved by the Institutional Animal Care and Use Committee of the Yonsei University College of Medicine (#2014-0275). All animal experimental protocols were performed in accordance with the relevant ethical guidelines and regulations. Immunocompetent, specific pathogen–free, 6-week-old female mice weighing 18–20 g (C57BL/6N) were used (Orient Bio, Seongnam, South Korea). Animals were rendered transiently neutropenic by injecting cyclophosphamide intraperitoneally (300 mg/kg body weight) in a volume of 0.2 mL 4 days before *A*. *baumannii* inoculation in the lung. The mice were anesthetized by intraperitoneal injection of 100 mg/kg ketamine and 10 mg/kg xylazine. Then, 1.2 mL/kg of a 5 × 10^8^ colony-forming units (CFU)/mL bacterial suspension was inoculated through the nose using a syringe^35^. After being kept in a vertical position for 4 min, the mice were maintained in a 30° decubitus position until regaining consciousness. Necropsy was performed on one mouse at 4, 24, and 48 h after the nasal inoculation. The entire lung was removed in a sterile fashion and evaluated pathologically, and the diagnosis of pneumonia was confirmed. Sterile lung specimens were fixed in 10% formalin and immersed in paraffin wax. Specimens were prepared in 5-μm cross sections and were examined under a light microscope after hematoxylin/eosin staining.

### Study groups

The mice with pneumonia were randomized into six groups of 15 mice each five treatment groups and a control group. Colistin was administered to the first group, fosfomycin to the second, minocycline to the third, the colistin/fosfomycin combination to the fourth, and the colistin/minocycline combination to the fifth. No antibacterial agent was administered to the mice in the control group. After the experiment, the mice were euthanized by CO_2_ inhalation.

### Treatment protocol

Treatments were initiated 4 h after nasal inoculation. The antibiotic agents were given by intraperitoneal injection at the following dosages: colistin, 20 mg/kg every 8 h^36^, fosfomycin, 100 mg/kg every 4 h^37^, and minocycline, 20 mg/kg every 12 h^38^. Colistin, fosfomycin, and minocycline were purchased from Sigma-Aldrich.

### Effects on lung bacterial loads

Bacteria in the lungs of three mice were counted 24 and 48 h after starting the antibiotic administration. To eliminate any antibiotic carry-over effect, mice in the treatment groups were euthanized at least 3 h after the last antibiotic dose. For quantitative bacteriological studies, the lungs were removed, weighed, and homogenized in 1 mL of saline. Tenfold dilutions were made, and 100-μL aliquots were plated on tryptic soy agar with 5% sheep blood plates for 24 h at 37 °C. Colonies were counted for each dilution and each animal. Experiments were performed in triplicate. The results are expressed as means ± standard deviation (SD) log_10_ CFU per gram lung at 24 and 48 h, and differences between groups were calculated as follows: mean of the treated group – mean of the control group. Bactericidal activity was defined as a ≥3 log_10_ decrease compared with the initial inoculum. Synergy was defined as a ≥2 log_10_ decrease in killing by the combination compared with the most active single drug alone^18^.

### Effects on survival

The survival rates of all mice at 24 and 48 h were recorded and compared among the treatment and control groups.

### Statistical analysis

All bacterial counts are presented as the mean ± SD. Student’s *t-*test was used to analyze inter-group differences in the bacterial counts. To compare mortality between groups, Fisher’s exact test was used. In all tests, differences were considered to be statistically significant when the *p*-value was <0.05.
